# Tuberculous Otitis Media With Cerebral Venous Thrombosis: A Rare and Challenging Diagnostic Case

**DOI:** 10.7759/cureus.54391

**Published:** 2024-02-18

**Authors:** Ayidh Alharthi, Ghayda A Alghamdi, Banan S Alghamdi, Ghadi S Alghamdi, Najla K Alzahrani

**Affiliations:** 1 Pediatric Neurology, King Fahad Hospital, Al Bahah, SAU; 2 College of Medicine, Al-Baha University, Al Bahah, SAU

**Keywords:** adolescent, tuberculoma, cerebral venous thrombosis, mastoiditis, tuberculous otitis media

## Abstract

Tuberculous otitis media is an uncommon cause of chronic suppurative infection affecting the middle ear and mastoid. Unfortunately, the signs and symptoms of tuberculous otitis media are very similar to those of non-tuberculous otitis media, which can make early diagnosis challenging. It is crucial to diagnose and treat the condition early to prevent damage to the ear and potential complications involving the central nervous system. We present a case of a 13-year-old Saudi girl who presented with a two-week history of headaches associated with photophobia. She had been complaining of ophthalmalgia, otalgia, and decreased hearing for one year. Physical examination revealed bilateral optic disc swelling with grade 3-4 papilledema, middle ear effusion, and bilateral hearing loss. CT scan showed sinusitis with right otomastoiditis and right petro-mastoiditis. MRI with magnetic resonance venography (MRV) revealed cerebral venous thrombosis. Tuberculosis screening by polymerase chain reaction (PCR) of a sputum sample and right ear effusion sample taken by tympanocentesis was done and it came back positive three weeks later. She was started on anti-tuberculous treatment, with clinical improvement observed over six months. Multiple factors contributing to the delay in diagnosis possibly included the lack of awareness about this ailment among doctors, leading to a low suspicion rate, variable clinical presentation, and diagnostic pitfalls.

## Introduction

Tuberculous otitis media (TOM) is an uncommon cause of chronic suppurative infection affecting the middle ear and mastoid [1]. While it was once believed to be a distinct disease, it is now recognized that tuberculosis may coexist with or develop in an ear that is already discharging [2]. Unfortunately, the signs and symptoms of TOM are very similar to those of non-TOM, which can make early diagnosis challenging. It is important to diagnose and treat the condition early, however, as this can help prevent damage to the ear and potential complications involving the central nervous system [3].

## Case presentation

A 13-year-old Saudi girl presented to the emergency room with a headache associated with photophobia for two weeks. She had been complaining of ophthalmalgia and otalgia for one year. The headache started two weeks prior to presentation and lasted for one hour in the forehead, accompanied by photophobia. Her ophthalmalgia was bilateral and intermittent, occurring once per month over the past year. Each episode lasted for two days and was partially relieved by over-the-counter paracetamol. The otalgia primarily affected her right ear and was initially diagnosed by an otolaryngologist as serous otitis media with no discharge. She was prescribed paracetamol and amoxicillin. Following this diagnosis, her pain became bilateral and was associated with tinnitus and decreased hearing, as well as neck pain. There is no history of fever, vomiting, loss of consciousness, or trauma. Additionally, the patient denied any chronic or recent respiratory symptoms including dry or productive cough, shortness of breath, and hemoptysis She had received routine pediatric vaccinations and has had normal development, except for left Erb's palsy since birth. Upon examination, she appeared ill and in pain but not pale, jaundiced, or cyanotic. The neurological examination was normal.

Ophthalmology consultation revealed bilateral optic disc swelling with grade 3-4 papilledema. The otorhinolaryngology consultation revealed right middle ear effusion, with intact bilateral tympanic membranes and normal bone trabecular of the mastoid and nasal sinuses. Pure tone average (PTA) audiometry indicated a mild bilateral conductive hearing loss, more prominent in the right ear.

**Figure 1:**
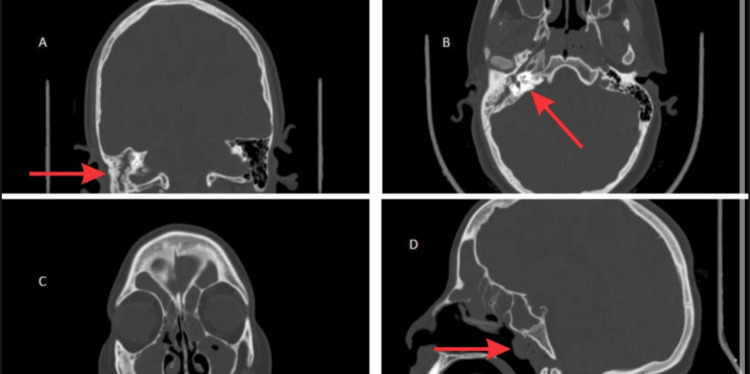
Non-contrast computerized tomography. Multiplanar bone window CT images show otomastoiditis (A), right petro-mastoiditis (B), adenoid hypertrophy (B,D), and gross pansinusitis (C,D).

Initial laboratory investigations showed leucocytosis of 18.40x10^9^ and neutrophilia of 83.70% with lymphocytopenia in the complete blood count (CBC). The erythrocyte sedimentation rate (ESR) was elevated at 105 mm/hour. Liver function tests, renal function tests, and bone profiles were all normal. An ear swab of the external canal was taken, but no growth was revealed. The activated partial thromboplastin time (APTT) was decreased with an elevated fibrinogen level. An initial non-contrast CT scan was done, which showed gross pansinusitis, right petro-mastoiditis and adenoidal hypertrophy (Figure 1).

**Figure 2:**
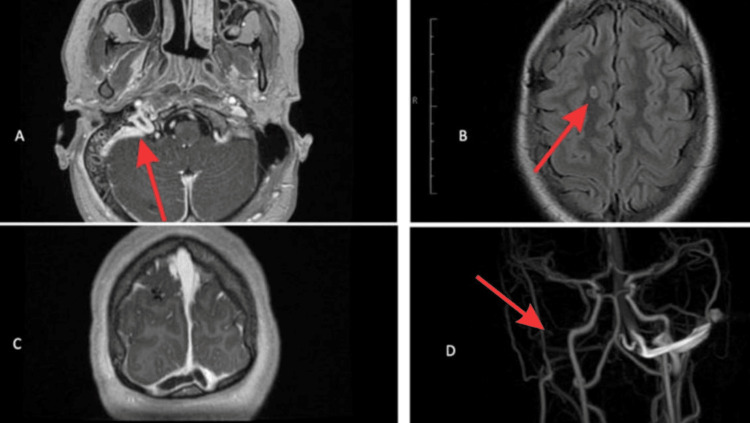
Contrast MRI findings. (A, C) indicate the presence of right ostomastoiditis complicated by thrombotic sequelae affecting the trochlea, as well as the proximal portion of the left transverse sinus (C), right transverse sinus, right sigmoid sinus, and the visible portion of the right internal jugular vein (A). The non-contrast FLAIR axial image (B) reveals the presence of an oval-shaped nodule with intermediate intensity in the right parietal subcortical/parasagittal white matter. Additionally, the MRV  (D) shows a lack of visualization of the right transverse and sigmoid sinus, as well as the internal jugular vein FLAIR: fluid-attenuated inversion recovery; MRV: magnetic resonance venography

Contrast MRI with magnetic resonance venography (MRV) was requested (Figure 2) revealing two solitary nodules, one in the right frontal subcortical white matter and the other in the right cerebellar hemisphere. The initial assumption was supratentorial and infratentorial infarcts from right-sided venous sinus thrombosis.

**Figure 3:**
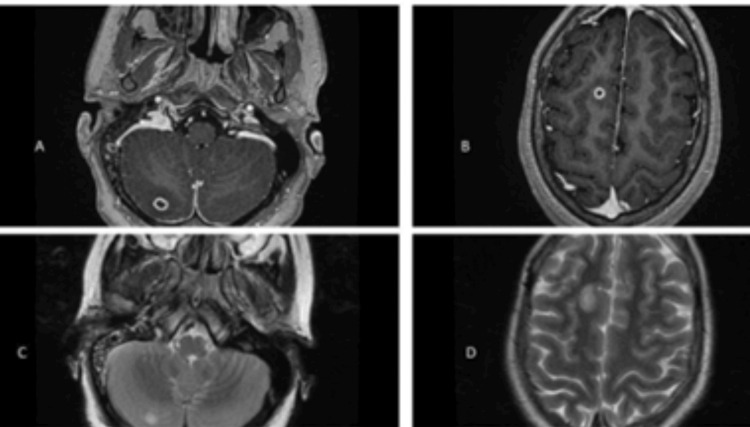
Contrast MRI with MRV brain showing an increase in the size of the right cerebellar and cerebral nodules (A,B), revealing well-defined peripheral ring enhancement (C,D).

After a week of admission, she started having episodes of fever of 38°C, and cerebral venous thrombosis at the occipital area secondary to mastoiditis was diagnosed. She was started on Clexane, vancomycin, and ceftriaxone. Three weeks later, a follow-up contrast MRI with MRV brain was done (Figure 3).

**Figure 4:**
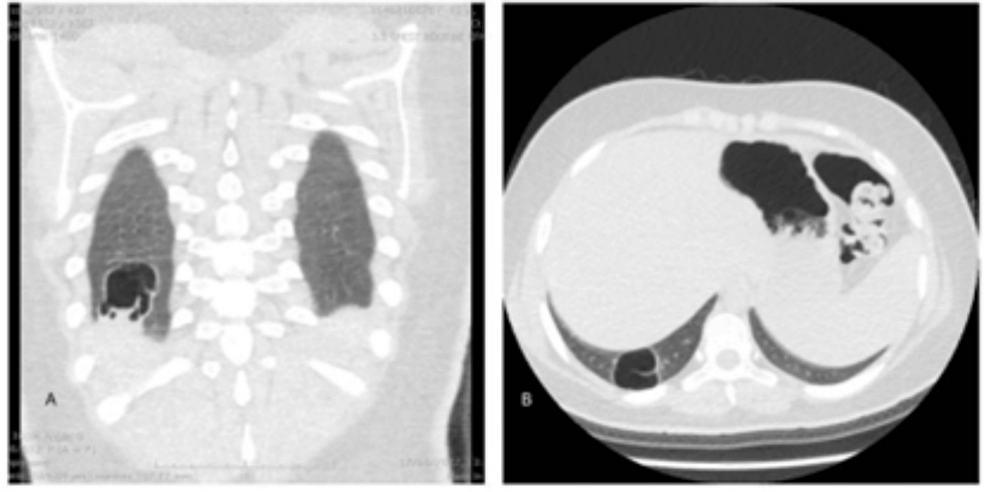
Chest CT showing a thick-walled large cavity in the right lower lobe with normal surrounding lung tissue.

Accordingly, a possible granulomatous condition was suspected. Therefore, purified protein derivative (PPD) test, sputum acid-fast bacilli staining, cerebrospinal fluid (CSF) analysis and culture, and TORCHES (toxoplasmosis, rubella cytomegalovirus, herpes simplex, and HIV) screening were done, and they all came back negative. Afterwards, tuberculosis screening by polymerase chain reaction (PCR) of a sputum sample and right ear effusion sample taken by tympanocentesis was done and it came back positive three weeks later. Chest CT was performed showing a thick-walled large cavity in the right lower lobe (Figure 4).

She was started on anti-tuberculous treatment (isoniazid, rifampicin, pyrazinamide, and levofloxacin) with additional supplementation such as pyridoxine and vitamin D3. Clexane was prescribed for two months. She started to show clinical improvement, as well as improvement in radiological findings and laboratory parameters over the next six months.

## Discussion

TOM is typically secondary to direct transmission from surrounding organs such as the lungs, larynx, throat, and nose, while the primary form is rare. However, its diagnosis is frequently delayed due to its rarity, slow course, or the absence of additional signs of current or prior infection in half of the cases [4]. Over the years, the clinical presentation of TOM has evolved, and it should be considered as a potential cause of chronic otitis media, especially in endemic areas, regardless of the presence of mastoiditis [5]. Diagnosing this condition remains challenging due to its non-specific symptoms and difficulties in confirming the diagnosis with microbiological tests of ear discharge [3]. Traditional characteristics of TOM, such as painless otorrhea and multiple tympanic membrane perforations, may not always be present, as seen in our case, further increasing the difficulty in reaching the correct diagnosis. Failure to suspect tuberculosis can lead to significant delays in diagnosis and complications.

In the majority of TOM cases, approximately 90% of patients experience hearing loss. However, the severity of deafness may not correlate with the extent of the disease, particularly in the acute phase [6]. Differentiating TOM from non-TOM can be challenging due to the similarity in symptoms and signs [7]. Israr et al. reported a case of an eight-year-old with cerebellar tuberculoma presenting with a headache, similar to the case under review [8]. Another case report demonstrated venous thrombosis in the internal jugular vein in a toddler, similar to our case, but as a result of disseminated tuberculosis [9].

In contrast to our study, there have been reported cases where the initial presentation includes painless purulent ottorhea accompanied the progressive deafness [10,11].

Notably, the clinical presentation of TOM exhibits remarkable variability, as evidenced by a retrospective clinical analysis encompassing diverse neuro-otologic manifestations documented in global literature, This comprehensive review highlights a spectrum of presentations, spanning from classical otological symptoms to intricate neurological sequelae associated with the condition [12]. Therefore, the challenge with diagnosing TOM lies in its clinical presentation, which often lacks specific features, necessitating a thorough evaluation and consideration of various differential diagnoses to confirm the condition accurately.

In this case report, the patient, a 13-year-old Saudi girl, presented to the emergency room with complaints of headache, photophobia, intermittent ophthalmalgia, and left ear otalgia for two weeks, suggesting the possibility of otitis media. Although partially resolved with antibiotics and analgesics, the patient developed bilateral pain with tinnitus and decreased hearing, along with neck pain. Otorhinolaryngology consultation revealed middle ear effusion and intact bilateral tympanic membranes. Pure tone audiometry confirmed bilateral hearing loss, more prominent in the left ear. Laboratory findings indicated leucocytosis, neutrophilia, lymphocytopenia, and elevated ESR. The APTT was decreased, and fibrinogen levels were elevated. Radiological assessments revealed right petro-mastoiditis, gross pansinusitis, and adenoid hypertrophy on non-contrast CT. Contrast MRI showed right ostomastoiditis complicated by venous thrombosis, involving the trochlea, right transverse sinus, right sigmoid sinus, the visualized part of the right internal jugular vein, and the proximal part of the left transverse sinus. The presence of two solitary nodules in the right frontal subcortical white matter and the right cerebellar hemisphere on MRV raised suspicion of a granulomatous condition, leading to further evaluation for infectious etiologies.

A definitive diagnosis of TOM requires microbiological and cultural tests, which in our case aided in the diagnosis of TOM with pulmonary TB complicated by mastoiditis and cerebral venous thrombosis. The patient demonstrated clinical improvement with the resolution of otalgia and hearing impairment as well as radiological improvement solely through the administration of four anti-tuberculosis therapies, including isoniazid, rifampicin, pyrazinamide, and levofloxacin, obviating the necessity for surgical intervention.

Long-term follow-up and regular audiological assessments are essential to ensure complete resolution, monitor hearing threshold, and identify any potential deterioration or complications.

## Conclusions

Diagnosing TOM presents challenges due to limited awareness among medical practitioners, its varied clinical presentation, and diagnostic complexities. Early suspicion of TOM, especially in cases unresponsive to standard antibiotics, is crucial. Swift confirmation through advanced diagnostic methods is essential to initiate tailored antituberculosis treatment promptly. Timely intervention not only prevents irreversible complications but also underscores the significance of maintaining a heightened index of suspicion and ensuring immediate diagnosis and treatment.

## References

[1] Hand JM, Pankey GA (2016). Tuberculous otomastoiditis. Microbiol Spectr.

[2] Reynaldo G, Carsantiningrum BC, Priyono H (2021). Tuberculous otitis media: A case report of hearing impairment in developing country. Oto Rhino Laryngologica Indonesiana.

[3] Sebastian SK, Singhal A, Sharma A, Doloi P (2020). Tuberculous otitis media -series of 10 cases. J Otol.

[4] Gupta N, Dass A, Goel N, Tiwari S (2015). Tuberculous otitis media leading to sequentialib bilateral facial nerve paralysis. Iran J Otorhinolaryngol.

[5] Yaniv E (1987). Tuberculous otitis media: a clinical record. Laryngoscope.

[6] Plester D, Pusalkar A, Steinbach E (1980). Middle ear tuberculosis. J Laryngol Otol.

[7] Kirsch CM, Wehner JH, Jensen WA, Kagawa FT, Campagna AC (1995). Tuberculous otitis media. South Med J.

[8] Khan DI, Anas M, Khan AA, Khan S (2019). Chronic headache: the only manifestation of cerebellar tuberculoma. Biosci Biotechnol Res Asia.

[9] Das S, Srinivasaraghavan R, Krishnamurthy S, Mahadevan S (2014). Internal jugular vein thrombosis complicating disseminated tuberculosis in a 2-year-old child. BMJ Case Rep.

[10] Lmekki S, Lecanu JB (2013). Tuberculosis of the middle ear and nasal passage: a case report. Int J Mycobacteriol.

[11] Bhatkar D, Utpat K, Desai U, Joshi JM (2017). Bilateral tuberculous otitis media: an unique presentation. Indian J Tuberc.

[12] Diplan Rubio JM, Alarcón AV, Díaz MP (2015). Neuro-otologic manifestations of tuberculosis. "The great imitator". Am J Otolaryngol.

